# The nuclear envelope localization of DYT1 dystonia torsinA-ΔE requires the SUN1 LINC complex component

**DOI:** 10.1186/1471-2121-12-24

**Published:** 2011-05-31

**Authors:** Michael T Jungwirth, Dhivya Kumar, Danielle Y Jeong, Rose E Goodchild

**Affiliations:** 1 Department of Biochemistry, Cellular and Molecular Biology, University of Tennessee, Knoxville, TN 37996, USA

**Keywords:** torsinA, LINC complex, AAA+ protein, Nuclear Envelope, SUN1, DYT1 dystonia

## Abstract

**Background:**

DYT1 dystonia is an autosomal dominant neurological condition caused by a mutation that removes a single glutamic acid residue (ΔE) from the torsinA (torA) AAA+ protein. TorA appears to possess a nuclear envelope (NE) localized activity that requires Lamina-Associated-Polypeptide 1 (LAP1), which is an inner nuclear membrane localized torA-binding partner. Although hypoactive, the DYT1 dystonia torA-ΔE isoform often concentrates in the NE, suggesting that torA-ΔE also interacts with an NE-localized binding partner.

**Results:**

We confirm that NE-localized torA-ΔE does not co-immunoprecipitate with LAP1, and find that torA-ΔE continues to concentrate in the NE of cells that lack LAP1. Instead, we find that variability in torA-ΔE localization correlates with the presence of the SUN-domain and Nesprin proteins that assemble into the LINC complex. We also find that siRNA depletion of SUN1, but not other LINC complex components, removes torA-ΔE from the NE. In contrast, the LAP1-dependent NE-accumulation of an ATP-locked torA mutant is unaffected by loss of LINC complex proteins. This SUN1 dependent torA-ΔE localization requires the torA membrane association domain, as well as a putative substrate-interaction residue, Y147, neither of which are required for torA interaction with LAP1. We also find that mutation of these motifs, or depletion of SUN1, decreases the amount of torA-WT that colocalizes with NE markers, indicating that each also underlies a normal NE-localized torA binding interaction.

**Conclusions:**

These data suggest that the disease causing ΔE mutation promotes an association between torA and SUN1 that is distinct to the interaction between LAP1 and ATP-bound torA. This evidence for two NE-localized binding partners suggests that torA may act on multiple substrates and/or possesses regulatory co-factor partners. In addition, finding that the DYT1 mutation causes abnormal association with SUN1 implicates LINC complex dysfunction in DYT1 dystonia pathogenesis, and suggests a gain-of-function activity contributes to this dominantly inherited disease.

## Background

DYT1 dystonia is a neurological disease characterized by prolonged, involuntary movements that develop in childhood or early adolescence, and occur in the absence of CNS pathology [1,2]. The disease is caused by an in-frame, loss-of-function mutation that removes a glutamic acid residue (ΔE) from torA [3,4]. TorA is a member of the AAA+ ATPase family (ATPases Associated with a variety of cellular Activities) that typically couple the energy released by ATP hydrolysis to conformational changes in binding partners. The structural changes induced by AAA+ proteins vary. However, in most cases, an oligomeric ring arrangement of AAA+ enzyme subunits pulls the binding-partner substrate into the central pore and, by doing so, 'stretches' or removes secondary structure from the substrate. This action often destabilizes an otherwise energetically favorable binding interaction, such as the presence of substrate in a protein complex, aggregate, or association with a lipid bilayer. There are many hundreds of AAA+ enzymes and substrates, and AAA+ enzymes are used in processes as diverse as DNA replication, membrane fusion, protein degradation and cytoskeletal movement [5-7].

Multiple studies and research groups have found that torA is an endoplasmic reticulum (ER) resident protein [8-10]. However, despite localization throughout the ER-system, torA loss specifically affects the NE subdomain and this suggests that torA AAA+ activity is targeted to a NE localized protein [4,11]. It is well established that torA interacts with the inner nuclear membrane protein, lamina-associated-polypeptide-1 (LAP1; TOR1AIP1) [12-15]. The importance of LAP1 is further underscored by the recent finding that LAP1 loss causes similar NE abnormalities to those seen in torA null cells [14]. In addition, the interaction between torA and LAP1 is stabilized by AAA+ domain mutations that typically inhibit ATP hydrolysis, such as the WalkerB box E171Q mutation in human torA [12,13,15]. Since the majority of AAA+ proteins interact with substrate in their ATP bound form, this stabilization suggests that LAP1 is a torA substrate. To date, however, the cellular functions of LAP1 remain unknown, no other luminal binding partners are identified, and LAP1 levels and subcellular localization appear unaffected by torA loss [4] - a surprising state of affairs for the predicted substrate of a physiologically important AAA+ protein. Other torA binding partners are also described, including the Nesprin proteins that are components of the LINC complex that couples the nuclear interior to cytoskeletal networks [16]. However, the relationship between these reported interacting partners, and the biochemical [12,13,15], genetic [14] and cell biologically [12,13] verified association between torA and LAP1 remains unclear.

Genetic analysis has demonstrated that the disease-associated torA-ΔE protein is expressed in DYT1 dystonia, but is hypoactive or inactive [4,10]. Consistent with these findings, recent studies demonstrated that ΔE appears to inhibit torA interaction with LAP1 and the homologous LULL1 membrane protein [13,15]. However, torA-ΔE can also concentrate in the NE [10,17], which suggests that ΔE stabilizes, rather than inhibits, interaction with an NE binding partner. The possibility that torA-ΔE displays enhanced NE-localized binding also argues against DYT1 dystonia being caused by a pure loss-of-function mutation. Furthermore, a gain-of-function activity is also consistent with the dominant nature of disease inheritance and that the torA-ΔE generating mutation is the sole causative mutation. Here we further examined torA-ΔE behavior in order to determine whether dysfunction of the mutant disease protein might contribute to DYT1 dystonia pathogenesis. We describe an association between torA-ΔE and the SUN1 inner nuclear membrane protein, and present data suggesting that SUN1 is also a normal torA binding partner. This abnormal ability of torA-ΔE provides more evidence for a gain-of-function action in DYT1 dystonia, and implicates the neurodevelopmentally important LINC complex dysfunction in disease pathogenesis.

## Results

**Figure 1:**
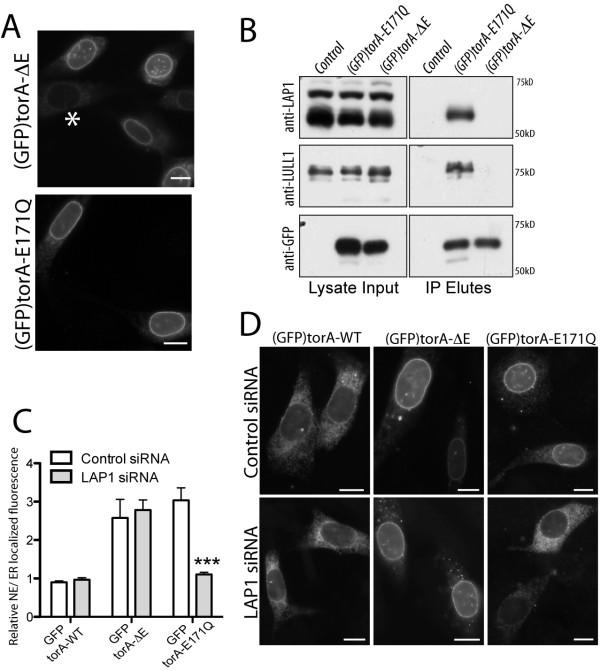
**TorA-ΔE does not interact with the NE-localized torA-E171Q binding partner, LAP1**. (A) (GFP)torA-ΔE and (GFP)torA-E171Q both concentrate in the NE of NIH-3T3 cells. The * symbol highlights a cell with ER-localized (GFP)torA-ΔE. Scale bars show 10 μm. (B) LAP1 and LULL1 co-immunoprecipitate with (GFP)torA-E171Q and not (GFP)torA-ΔE. Left panels show levels of LAP1, LULL1 and (GFP)torA in 5% of lysate input collected from control, (GFP)torA-E171Q or (GFP)torA-ΔE stably expressing NIH-3T3 cells. Anti-LAP1 (top row) detects the alternatively spliced LAP1 isoforms [14,43] and anti-LULL1 (middle row) detects 75 kD LULL1. Right panels show levels of LAP1, LULL1, or GFP that immunoprecipitate with anti-GFP in buffer containing 2 mM ATP and 0.5 mM ADP. Note: mild elution conditions were used to prevent release of captured anti-GFP IgG molecules. (C) LAP1 depletion specifically affects (GFP)torA-E171Q localization. Columns show the mean and s.e.m. for ratios of NE/ER localized GFP fluorescence from n > 25 NIH-3T3 cells co-transfected with scrambled or LAP1 siRNA duplexes and (GFP)torA isoforms. *** symbol indicates that LAP1 depletion significantly reduces the (GFP)torA-E171Q NE/ER fluorescence ratio (p < 0.001; Two-Way ANOVA with Bonferroni post-hoc analysis). (D) (GFP)torA-ΔE remains NE-localized in LAP1 depleted cells, while (GFP)torA-E171Q redistributes into the ER. Images show anti-GFP labeling of NIH-3T3 cells co-transfected with (GFP)torA isoforms and control (upper row) or LAP1 siRNA duplexes (lower row). Scale bars show 10 μm.

The majority of NE membrane proteins are localized and immobilized in this ER subdomain by binding to the nuclear lamina [18,19]. Although torA is a luminal protein, torA-E171Q and torA-ΔE concentrate in the NE of several cell lines, and each diffuses more slowly than ER-localized torA [12], which suggests that both isoforms interact with a lamina-associated NE membrane protein, such as LAP1. We examined the LAP1-binding of human torA-ΔE and torA-E171Q using mouse NIH-3T3 cells where both isoforms similarly localize in the NE (Figure 1A), whereas this is previously assessed using cell lines where torA-ΔE is predominantly ER-localized [15,20]. However, we also find that LAP1 specifically co-immunoprecipitates with (GFP)torA-E171Q, but not (GFP)torA-ΔE (Figure 1B, top row), despite similar expression and capture of both GFP-tagged torsinA isoforms (Figure 1B, bottom row). We also compared (GFP)torA-E171Q and (GFP)torA-ΔE binding to the ER-localized torA binding partner, LULL1. Again, we find no detectable co-immunoprecipitation of LULL1 with (GFP)torA-ΔE, although LULL1 is co-captured by (GFP)torA-E171Q immunoprecipitation (Figure 1B, middle row). Thus, it appears that the E171Q and ΔE mutations differentially affect binding to the two verified torA binding partners, even in cell systems where both mutations cause torA to accumulate in the NE.

Co-immunoprecipitation of LAP1 with torA requires that binding is maintained after detergent solubilization removes both proteins from the ER membrane. It is therefore possible that ΔE inhibits this assessment of torA binding to LAP1, while interaction *in vivo *continues to underlie torA-ΔE localization in the NE, and this could be exacerbated because we are examining human torA in a mouse cell line. Consequently, we next examined whether LAP1 is important for the *in vivo *localization of torA-ΔE. To semi-quantitatively assess protein localization *in vivo*, we measured the relative amount of NE and ER-localized protein in individual cells. This method finds ratios of NE/ER localized fluorescent signal above one for the inner nuclear membrane proteins of LAP1, LAP2 and emerin (the mean ratio ± standard error (s.e.m.) of NE/ER localized LAP1 fluorescence is 2.86 ± 0.05 (*n *= 25), LAP2 is 1.59 ± 0.05 (*n *= 10), and emerin is 1.63 ± 0.08 (*n *= 23)). In contrast, ER-resident proteins that do not accumulate in the NE display relative NE/ER fluorescence ratios below one (mean ± s.e.m. ratio of NE/ER calreticulin fluorescence is 0.94 ± 0.04, *n *= 12, and protein disulphide isomerase (PDI) is 0.79 ± 0.19, *n *= 25).

We subsequently co-transfected NIH-3T3 cells with (GFP)torA-WT, (GFP)torA-ΔE, or (GFP)torA-E171Q, together with scrambled control or LAP1 siRNA duplexes. In agreement with previous reports where LAP1 was required for the NE accumulation of (GFP)torA-E171Q [14,21], the semi-quantitative analysis detected that LAP1 depletion significantly reduces the mean ratio of NE/ER localized (GFP)torA-E171Q fluorescence (Figure 1C). However, in contrast, LAP1 knock-down did not affect the ratio of NE/ER (GFP)torA-ΔE fluorescent signal (Figure 1C). Qualitative analysis similarly highlights that (GFP)torA-ΔE is strongly concentrated in the NE of LAP1 depleted cells (Figure 1D; central panels), while the absence of LAP1 inhibits the NE-accumulation of (GFP)torA-E171Q, which is instead predominantly ER-localized (Figure 1D; right column). Although torA-WT is previously shown to biochemically associate with LAP1 [13,15], and LAP1 overexpression increases the amount of NE-localized (GFP)torA-WT [12], we failed to detect LAP1-induced change in (GFP)torA-WT localization. However, this may be due to the insensitivity of our measurements, rather than absence of *in vivo *interaction between torA-WT and LAP1.

### LINC complex components are required for torA-ΔE localization in the NE

This lack of interaction between torA-ΔE and LAP1 reveals that a distinct mechanism underlies the NE-accumulation of the disease-associated torA isoform. Furthermore, although (GFP)torA-ΔE concentrates in the NE of the majority of NIH-3T3 and BHK21 cells, we consistently observe a minority of cells where (GFP)torA-ΔE is diffusely distributed through the ER (Figure 1A, where the * symbol highlights a cell with ER-localized (GFP)torA-ΔE), and this variability does not correlate with levels of LAP1 (not shown). We hypothesized that this inconsistency in torA-ΔE localization might reflect the variable presence of a NE-localized torA-ΔE binding partner and we proceeded to examine the subcellular localization of several NE membrane proteins. We focused on the LINC complex proteins that couple the nucleus with cytoskeletal networks, as these have previously been associated with torA [16,20]. The LINC complex is formed in the NE lumen between inner nuclear membrane SUN-domain proteins and outer nuclear membrane Nesprins, and we find that NIH-3T3 cells express both of the functionally homologous SUN1 and SUN2 proteins [22,23]. We also detect NE-localized Nesprin2 in NIH-3T3 cells, although Nesprin1 is absent [24] and anti-Nesprin3 signal appears Golgi-localized (Additional File 1, Figure S1), thus indicating that Nesprin2 is the predominant Nesprin protein of NIH-3T3 cells.

**Figure 2:**
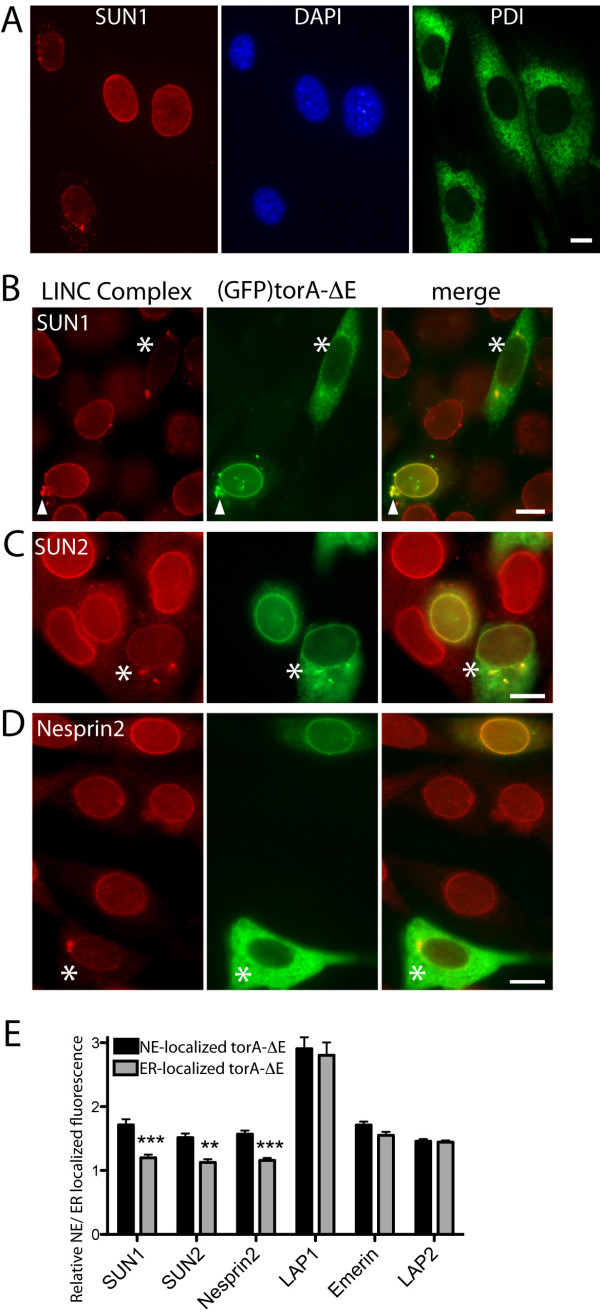
**TorA-ΔE co-localizes with LINC complex components**. A) SUN1 localization in the NE varies between individual NIH-3T3 cells. Panels show anti-SUN1 (red), DAPI labeling of DNA, anti-PDI labeling of the ER (green) in sub-confluent, unsynchronized NIH-3T3 cells. Scale bars show 10 μm. (B - D) Positive correlation between the localization of LINC complex components and transfected (GFP)torA-ΔE. Left panels (red) show (B) anti-SUN1, (C) anti-SUN2, and (D) anti-Nesprin2 labeling of NIH-3T3 cells. Middle panels (green) show the subcellular localization of transfected (GFP)torA-ΔE. * symbols highlight that cells with ER-localized (GFP)torA-ΔE also lack NE-localized LINC proteins. White arrowheads highlight that transfected (GFP)torA-ΔE also colocalizes with SUN1 outside the NE. (E) Ratios of NE/ER localized anti-SUN1, SUN2 and Nesprin2 fluorescence are higher in cells with NE-localized (GFP)torA-ΔE. Columns show the mean and s.e.m. of the relative NE/ER localized anti-SUN1, SUN2, Nesprin2, LAP1, emerin and LAP2 fluorescence from n > 15 cells. Black columns show the ratios of NE/ER fluorescence from cells with predominantly NE-localized (GFP)torA-ΔE, while grey columns show ratios from cells with ER-localized (GFP)torA-ΔE. ***, ** indicate that the mean ratio of NE/ER fluorescence is significantly (p < 0.001, 0.01) different between cells with NE- or ER-localized (GFP)torA-ΔE (Two-Way ANOVA with Bonferroni post-hoc analysis).

While the majority of NIH-3T3 cells possess NE-localized anti-SUN1, anti-SUN2 and anti-Nesprin2 immunoreactivity, we also observe some interphase cells with minimal amounts of NE-localized fluorescent signal, or punctate anti-LINC component immunoreactivity outside of the NE (Figure 2A shows SUN1 localization). In contrast, the inner nuclear membrane proteins of emerin, LAP1 and LAP2 are consistently NE-localized in interphase cells (not shown), suggesting that this variability is LINC complex specific. We consequently examined whether (GFP)torA-ΔE localization correlates with the variability in LINC complex protein localization and found that cells with NE-localized anti-SUN1, anti-SUN2 or anti-Nesprin2 labeling also display NE-localized (GFP)torA-ΔE (Figure 2B-D). In contrast, (GFP)torA-ΔE is diffusely distributed through the ER system in cells that lack NE-localized LINC complex components, or when these were present at low levels (Figure 2B-D, cells highlighted by * symbols). Furthermore, we often also observed that (GFP)torA-ΔE co-localized with SUN1-immunoreactive puncta outside the NE (Figure 2B; white arrow head). We subsequently confirmed this qualitative association between (GFP)torA-ΔE and LINC complex localization by examining the ratio of NE/ER localized anti-SUN1, anti-SUN2 and anti-Nesprin2 fluorescence in cells with either NE- or ER-localized (GFP)torA-ΔE. This analysis found that the ratios of NE/ER localized LINC complex components were significantly higher for cells with NE-localized (GFP)torA-ΔE (Figure 2E, black columns), compared with cells where (GFP)torA-ΔE is predominantly present in the main ER (Figure 2E, grey columns). In contrast, we found no correlation between torA-ΔE localization and the relative NE/ER levels of LAP1, emerin or LAP2 (Figure 2E).

**Figure 3:**
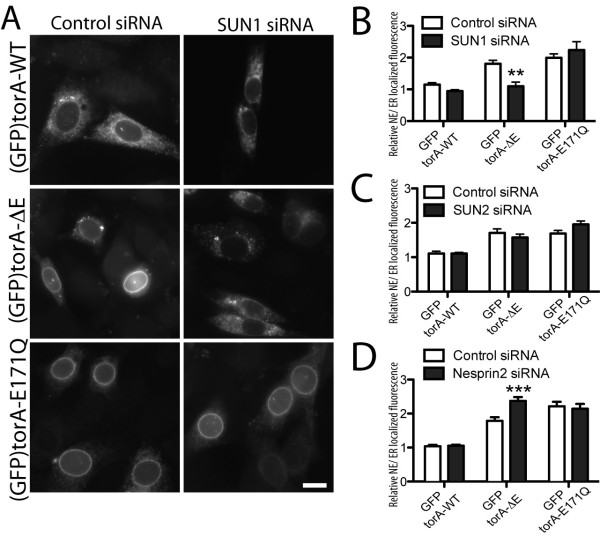
**TorA-ΔE localization in the NE requires SUN1**. (A) SUN1 depletion selectively inhibits the NE-localization of (GFP)torA-ΔE. Images show anti-GFP labeling of NIH-3T3 cells co-transfected with (GFP)torA isoforms and control (left panels) or SUN1 siRNA duplexes (right panels). Scale bar shows 10 μm. (B - D) Only SUN1 siRNA reduces the ratio of NE/ER localized (GFP)torA-ΔE fluorescence. Columns show the mean and s.e.m. of the relative NE/ER GFP fluorescence from n > 25 NIH-3T3 cells transfected with (GFP)torA isoforms alongside (B) SUN1, (C) SUN2, or (D) Nesprin2 siRNA duplexes, or a sequence matched scrambled control siRNA duplex. The ** and *** symbols indicate a significant (p < 0.01, < 0.001, respectively) difference between control and siRNA treated cells. (Two-Way ANOVA with Bonferroni post-hoc analysis.)

We next examined whether any LINC complex components are required for torA-ΔE to localize in the NE. We co-transfected NIH-3T3 cells with (GFP)torA isoforms alongside scrambled control siRNA duplexes, or SUN1, SUN2, or Nesprin2 siRNA duplexes that were verified to reduce expression of each LINC complex component (Additional File 1, Figure S1B - D). This revealed that SUN1 is required for (GFP)torA-ΔE to concentrate in NE, and we find ER-localized (GFP)torA-ΔE, or a punctate distribution of (GFP)torA-ΔE, in cells lacking SUN1 (Figure 3A middle row). As expected, this change in subcellular localization is reflected by a significant decrease in relative NE/ER (GFP)torA-ΔE fluorescence between control and SUN1 depleted cells (Figure 3B). In contrast, (GFP)torA-E171Q continues to concentrate in the NE of SUN1 siRNA treated cells (Figure 3A, bottom row) and SUN1 siRNA does not appear to alter the relative NE/ER localization of (GFP)torA-E171Q (Figure 3B). This suggests that SUN1 loss specifically affects torA-ΔE without perturbing all NE-localized interactions. We do not observe gross differences in the localization of any torA isoform after SUN2 or Nesprin2 depletion (not shown). We also determined that the relative NE/ER fluorescence of both (GFP)torA-ΔE and (GFP)torA-E171Q remains high in SUN2 siRNA transfected cells (Figure 3C). Surprisingly, this semi-quantitative analysis identified that Nesprin2 depletion increases the ratio of NE/ER localized (GFP)torA-ΔE (Figure 3D). Thus, although Nesprins are identified as torA-binding proteins [16], it appears that removing Nesprin2 may increase the number of NE-localized torA-ΔE binding sites.

### TorA-WT and torA-ΔE localization in the NE requires motifs that are not involved in torA-E171Q interaction with LAP1

**Figure 4:**
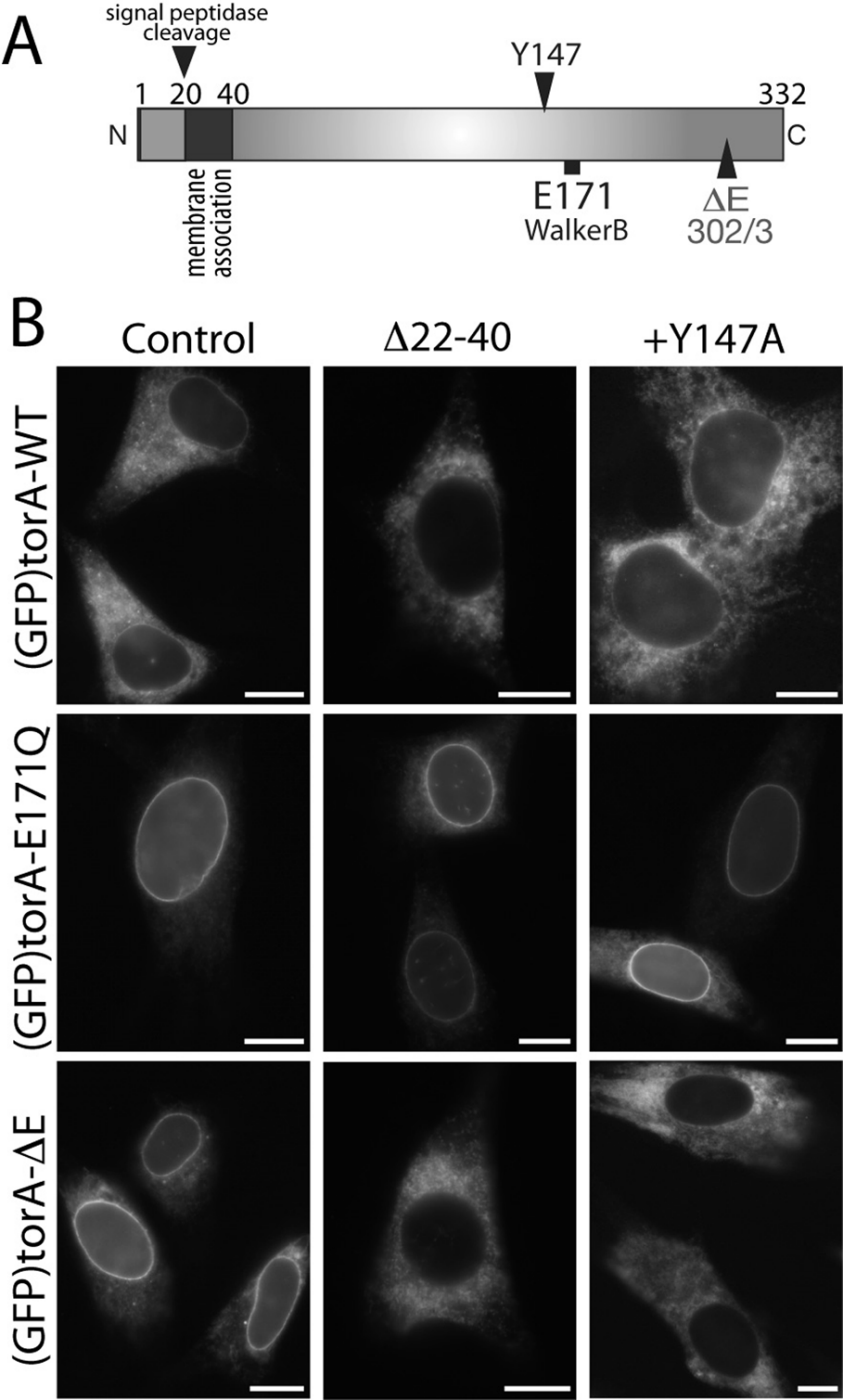
**A putative membrane association domain and pore-lining residue are required for torA-ΔE localization in the NE**. (A) Schematic of torA structure showing the location of amino-terminal ER targeting sequence, putative membrane association domain, together with the Y147, E171 and E302/3 (mutated by ΔE) residues. (B) Images show anti-GFP labeling of BHK21 cells expressing (left panels) "control" (GFP)torA-WT, (GFP)torA-ΔE or (GFP)torA-E171Q proteins, or (central column) these torA isoforms lacking residues 22 - 40 ("Δ22-40"), or (right column) torA isoforms containing the Y147A mutation ("+ Y147A"). Scale bars show 10 μm.

These observations suggest that an association exists between torA-ΔE and the SUN1 LINC complex component, and therefore that SUN1, rather than LAP1, may be an NE partner of torA-ΔE. We subsequently examined whether these two putative torA binding interactions require distinct torA domains or residues. We first investigated the importance of the torA membrane association domain that immediately follows the cleaved ER targeting sequence [20,25] (Figure 4A). We transfected NIH-3T3 cells with either full-length (GFP)torA isoforms, or torA isoforms that lacked residues 22 to 40 (Δ22-40) that includes the membrane-association domain. We found that (GFP)torA-E171Q lacking residues 22-40 continues to accumulate in the NE (Figure 4B middle row), indicating that this domain is not required for LAP1 binding, as is consistent with previous study of the torA - LULL1 interaction [20]. In contrast, Δ22-40 inhibits the NE-concentration of (GFP)torA-ΔE, and we fail to observe any cells with NE-localized (GFP)torA-ΔE when residues 22-40 are removed (Figure 4B). We also observed that Δ22-40 appeared to decrease the amount of NE-localized torA-WT (Figure 4B top row). We therefore semi-quantitatively examined (GFP)torA-WT localization, which revealed that introduction of Δ22-40 caused a significant (p < 0.001, Students Two-Tailed T-Test) decrease in the relative amount of NE/ER localized (GFP)torA-WT fluorescence from a mean ± s.e.m. of 1.15 ± 0.03 to 0.98 ± 0.03 (*n *= 25). Thus, the membrane association domain of torA is both essential for the SUN1-dependent NE accumulation of torA-ΔE, and appears to play a role in a torA-WT localization, despite not affecting torA interaction with LAP1 or LULL1.

We also explored whether other torA motifs are important for torA localization in the NE. AAA+ enzymes typically alter substrate conformation using aromatic amino-acid residues that line the pore of the assembled AAA+ ring structure. Work with model AAA+ proteins has suggested that these residues interact with substrate and undergo 'lever-like' movements to pull substrates into the central pore [26-28]. We identified a torA tyrosine residue (Y147 in human torA) that lies in the approximate position of other AAA+ substrate-binding tyrosine residues. Furthermore, this residue is conserved between all vertebrate and invertebrate torA homologs (torsin family proteins [21]), suggesting a functional importance in torsin enzyme activity. With the hypothesis that this residue may be specifically important for torA binding with substrate, we compared how mutation (Y147A) affects (GFP)torA-ΔE localization in the NE compared with the LAP1-dependent NE-localization of (GFP)torA-E171Q. As with residues 22-40, we observed that the Y147A mutation specifically inhibits the NE-localization of (GFP)torA-ΔE, while (GFP)torA-E171Q continues to accumulate in the NE (Figure 4B, right column). We again also observed an apparent loss of NE-localized (GFP)torA-WT fluorescent signal and therefore semi-quantitatively examined how Y147A affects (GFP)torA-WT localization. This revealed that the Y147A mutation significantly reduces (p < 0.05, Students Two-Tailed T-Test) the ratio of NE/ER (GFP)torA-WT fluorescence from a mean ± s.e.m. of 0.98 ± 0.02, to 0.90 ± 0.02 (*n *= 45). Thus, the Y147 residue is required for both the NE-concentration of torA-ΔE, as well as for torA-WT to localize in the NE.

### SUN1 depletion removes torA-WT from the NE

**Figure 5:**
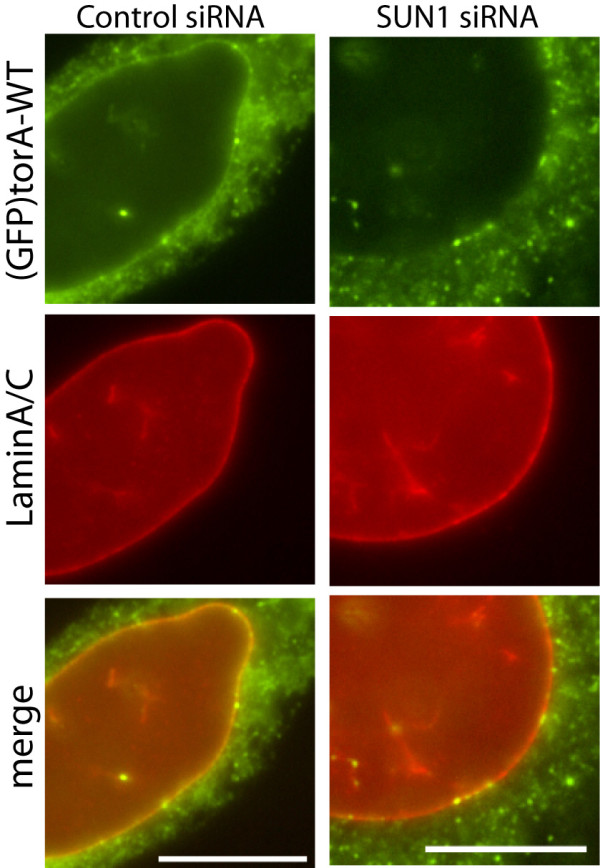
**SUN1 depletion also reduces NE-localized torA-WT**. The (GFP)torA-WT normally present in the NE of NIH-3T3 cells is reduced by SUN1 siRNA treatment. Images show anti-GFP labeling of NIH-3T3 cells expressing (GFP)torA-WT (green) and transfected with control (left) or SUN1 siRNA duplexes (right). Anti-LaminA/C labeling (red) highlights the position of the NE and lower panels show the merge (yellow) of (GFP)torA-WT and NE-marker signals, with less yellow signal present in the cell lacking SUN1. Scale bars show 10 μm.

The importance of 22-40 and Y147 for WT and torA-ΔE localization in the NE suggests that these torA isoforms form a similar, LAP1-independent, binding interaction. Consistent with this hypothesis, we often observed that SUN1 depletion reduced the small amount of NE-localized (GFP)torA-WT fluorescence present in most cells [10,29] (Figure 5). Our initial analysis found that SUN1 siRNA did not significantly decrease the ratio of NE/ER localized (GFP)torA-WT fluorescence. However, since a proportion of control treated cells also lack NE-localized SUN1 (Figure 2A shows variability in SUN1 levels), we hypothesized that our approach may have yielded a false-negative result. Consequently, we reassessed the ratio of NE/ER localized (GFP)torA-WT fluorescence in individual control siRNA transfected cells that we verified possessed SUN1, and compared this with cells that were SUN1 siRNA treated and verified to lack SUN1. This analysis found that the control ratio of NE/ER (GFP)torA-WT fluorescence in SUN1-containing cells (mean ± s.e.m. of 1.03 ± 0.02 (*n *= 34)) is significantly reduced (p < 0.0001, Students Two Tailed T-Test) to 0.89 ± 0.02 (*n *= 41) when SUN1 siRNA transfection depletes cellular SUN1. Thus, it appears that some torA-WT is normally SUN1-associated *in vivo*, which suggests SUN1 is a normal torA partner, as well as the protein responsible for the abnormal NE accumulation of disease-associated torA-ΔE.

## Discussion

LAP1 has been consistently identified as a NE-localized binding partner of torA [12,14,15,20], and it appears that LAP1 is required for NE-localized torA activity [14]. However, we find that the NE localization of both torA-WT and torA-ΔE require torA-motifs that are unimportant for the LAP1 interaction. Furthermore, we show that SUN1 depletion removes torA-WT and torA-ΔE from the NE, while LAP1 is not required for torA-ΔE to concentrate in the NE. Therefore, although LAP1 is the confirmed binding partner for ATP-bound torA, LAP1 is neither the sole NE-localized torA interacting protein nor the partner of disease-associated torA-ΔE.

The multiple differences between torA-E171Q and torA-ΔE reveal that distinct mechanisms underlie their superficially similar ability to concentrate in the NE lumen [13,15]. While it appears that the E171Q mutation traps torA in an ATP-bound state, the mechanism by which ΔE affects torA remains unclear. A previous genetic analysis found that torA-ΔE cannot compensate for torA loss, therefore revealing that this isoform is hypoactive or inactive [4]. Biochemical analysis has also demonstrated that torA-ΔE may fail to bind ATP, or perhaps fail to undergo a structural rearrangement on ATP-binding [15], either of which may explain why torA-ΔE cannot form the ATP-dependent interaction with LAP1 or LULL1 [13,15]. Despite these inhibitory effects, our data now highlight that ΔE does not generate a 'dead' torA isoform that is incapable of all binding interactions. Instead, the abnormal association between torA-ΔE and SUN1 suggests that ΔE 'traps' torA in a specific conformation that is distinct to the ATP-bound state. This finding also provides further evidence that the DYT1 ΔE mutation has a gain-of-function activity, in addition to producing the previously characterized impairment of torA function.

Our data indicate that torA associates with the SUN1 LINC complex component, and that the DYT1 mutation abnormally promotes or stabilizes this interaction. SUN1 is an inner nuclear membrane component of the LINC complex that couples the nuclear interior to cytoskeletal networks. SUN1 has a nucleoplasmic domain that mediates interaction with lamins, an extended membrane spanning hydrophobic region, and an approximately 50 kD luminal domain that appears to mediate both SUN-protein multimerization and interaction with KASH-domains of outer nuclear membrane Nesprin proteins [30-33]. There is loss-of-function evidence for functional redundancy between SUN1 and the similarly widely expressed and homologous SUN2 [22,23]. It is therefore surprising to find that SUN1 is specifically required for torA localization in the NE. We cannot rule out that SUN1 is selectively required because NIH-3T3 cells predominantly rely on SUN1, or that SUN1 siRNA more efficiently depletes SUN1 from the NE. However, there are reported differences in SUN1 and SUN2 characteristics [20,22,24,34], and our finding that SUN1 is selectively important for torA localization provides further evidence that these proteins have distinct, as well as overlapping cellular roles.

We do not resolve whether torA interacts directly or indirectly with SUN1. However, there is evidence against the possibility that SUN1 loss causes general NE disruption that removes torA by a highly indirect mechanism. Several previous studies have shown that NE structure and LINC complex function are largely normal in the absence of SUN1, and that combined SUN1 and SUN2 loss is required to perturb NE morphology [23,30,35,36]. Consistent with these findings, we observe that the NE-localization of LAP1-associated torA-E171Q is undisturbed by SUN1 loss, and that other inner nuclear membrane proteins are normally localized in cells lacking SUN1 (not shown). Furthermore, we also find that torA-ΔE colocalizes with SUN1 in puncta that lie outside of the NE. Thus, while it is possible that torA indirectly associates with SUN1, any such indirect interaction would be mediated by a protein or proteins that are also tightly coupled to SUN1. Unfortunately, we were unable to biochemically verify that a direct or indirect interaction exists between torA and SUN1. Like many inner nuclear membrane proteins, SUN1 solubilization requires that ionic detergents disrupt binding to the nuclear lamina and chromatin [31]. This treatment necessarily also impairs luminal interactions and a failure to detect biochemical association does not preclude that SUN1 and torA interact *in vivo*. Furthermore, despite our negative findings with anti-GFP immunoprecipitation of (GFP)torA isoforms, one previous report described that nesprin KASH-domains co-immunoprecipitate with anti-torA antibodies [16], which provides general support for an interaction between torA and a LINC complex component. However, our finding that Nesprin2 depletion increases, rather than decreases, the amount of NE-localized torA-ΔE, indicates that this previous result reflects an indirect, rather than direct, interaction between torA and nesprins. It is unclear why Nesprin2 loss increases torA-ΔE localization in the NE. However, an increased number of torA-ΔE binding sites, perhaps caused by compensatory upregulation of other LINC components, could account for both our observations and the previous association between torA and the nesprin KASH-domain.

Our findings support a model where torA interacts with at least two different NE proteins [16]. The existence of additional NE-localized partners suggests that torA either operates on multiple substrates and/or that some identified torA interacting partners have a regulatory function and are not subject to torA AAA+ activity. Finding that the SUN1-dependent localization requires a putative substrate interaction residue [26,28], Y147, suggests that SUN1 or the LINC complex is a substrate affected by torA AAA+ activity. Surprisingly, this also suggests that Y147A destabilizes an interaction that is distinct to the ATP-bound torA state that associates with LAP1 and LULL1. This insensitivity of LAP1 and LULL1 binding raises the possibility that these proteins are not torA substrates. Furthermore, these data also suggest that torA could operate using an atypical biochemical AAA+ mechanism; a hypothesis that is supported by the presence of a non-canonical nucleotide binding motif in the torA AAA+ domain that appears to convey preferential binding to ADP, rather than ATP [37].

The possibility that torA activity modifies the LINC complex is also supported by two previous reports. In one study, torA loss appeared to remove the Nesprin3 LINC complex component from the NE, suggesting that torA normally maintains intact LINC complexes [16]. In contrast, a separate study made the reverse observation, and found that overexpression of the LULL1 torA-binding partner appeared to induce a NE-localized torA activity that removed SUN2 and Nesprin2 [20]. This LULL1-activated torA was strongly concentrated in the NE of cells that lacked SUN2 and Nesprin2, which supports our finding that SUN1 is important for the NE retention of torA. Furthermore, since AAA+ activity often disassembles otherwise stable protein complexes [6,38], this data is also consistent with torA dissociating SUN1 binding interactions to release SUN1-associated proteins from the NE lumen. The relationship between the LULL1-overexpression paradigm and physiological torA function is unclear, and there are some AAA+ proteins that promote protein complex formation or remodeling, rather than disassembly [39]. Therefore, a difference between the actions of physiological levels of torA activity, versus overactive torA enzymes, could explain the discrepancy between these two studies.

While our study emphases a relationship between torA and the LINC complex, it is also clear that LAP1 has a central role in torA function and, notably, LAP1 and torA gene knock-out result in the same NE membrane abnormalities [14]. Although this association does not resolve whether LAP1 activity is upstream (an essential torA regulator/co-factor) or downstream (a substrate) of torA activity, it is clear that LAP1 is the partner of the ATP-bound form of torA that is typically the substrate-associated state of a AAA+ protein [13,15]. There are examples of promiscuous AAA+ enzymes that operate on several distinct substrate proteins, and it is possible that torA is a multi-functional AAA+ protein that operates on both LAP1 and the LINC complex. Furthermore, since adapter proteins often regulate the substrate selection of multifunctional AAA+ proteins [40], the importance of Y147, residues 22-40, and the effect of ΔE, could be explained if these motifs affect torA interaction with an adapter that promotes the LINC complex association over LAP1 binding.

Our study is performed using cells that also express torA. It is therefore possible that these endogenous torA-WT subunits play a role in the association between (GFP)torA-ΔE and SUN1. Nevertheless, mixed torA-ΔE and torA-WT expression also occurs in DYT1 dystonia, and it is demonstrated that torA abnormally concentrates in the NE of DYT1 dystonia cells, as well as neurons ectopically expressing torA-ΔE [10,29]. Thus, our findings suggest that the LINC complex of DYT1 dystonia neurons is abnormally associated with torA-ΔE. There are several mechanisms by which an abnormal association between torA-ΔE and SUN1 could negatively impact cell function. Firstly, torA-ΔE occupation of binding sites could prevent functional torA enzymes from accessing the LINC complex, and therefore inhibit torA activity in the event that this normally modifies the LINC complex. Secondly, there is evidence that torA-WT and torA-ΔE co-oligomerize, and that the abnormal SUN1-association of torA-ΔE is conferred to co-expressed torA-WT [10]. This could result in torA-WT sequestration away from other molecular targets of torA AAA+ activity. A third possibility is that abnormal association with torA-ΔE directly impacts LINC complex assembly or function, independent of torA-WT activity. These mechanisms are not mutually exclusive, and it is possible that a combination of such loss-of-function and/or gain-of-function torA-ΔE actions underlie why DYT1 dystonia is dominant and the torA-ΔE generating mutation is the only identified cause of this disease.

Our study did not identify grossly abnormal LINC complex component localization in cells transfected to express (GFP)torA-ΔE. However, our assessment did not examine whether physiological torA-ΔE expression impacts LINC complex components as these are utilized during particular cellular behaviors, such as nuclear movement [23,24]. It is unlikely that torA-ΔE expression completely ablates LINC complex activity, as genetic deletion of the complex in mice results in severe neurodevelopmental abnormalities [23], compared with undetectable or limited neuropathology in human DYT1 dystonia patients and heterozygous torA-ΔE expressing mice [41]. However, given the importance of the LINC complex for nervous system development [23], it is conceivable that torA-ΔE driven abnormalities in LINC complex mediated events impact neuronal development to generate the circuit abnormalities that are thought to underlie the debilitating twisting movements of DYT1 dystonia [42].

## Conclusions

We have demonstrated an association between the DYT1 dystonia protein, torA-ΔE, and the SUN1 component of the LINC complex. We also find evidence that SUN1 is a normal torA partner, which therefore suggests that the ΔE mutation stabilizes a normally transient torA binding interaction. These findings raise the possibility that torA-ΔE may impact the LINC complex, and therefore implicate LINC complex dysfunction in DYT1 dystonia.

## Methods

### Cell culturing and transfection

We obtained NIH-3T3 and BHK21 cells from ATCC and cultured these under recommended conditions. Transfections were performed for 3 hours with cells plated onto collagen-coated coverglasses in 24 well plates using Plus Reagent with either Lipofectamine or Lipofectamine LTX. siRNA transfection of NIH-3T3 cells was performed using 25 pmol dsRNA and RNAiMAX reagent. Dual plasmid and RNA transfection of ~ 30% confluent NIH-3T3 cells was performed using 200 ng DNA, 25 pmol dsRNA and Lipofectamine 2000. All transfection reagents were purchased from Invitrogen and used according to the manufacturers recommendations except as noted above. We generated (GFP)torA stably expressing NIH-3T3 lines by placing plasmid transfected cells under selection with 1 mg/ml G418 (Fisher Scientific) until patches of cells were visible. Individual colonies were collected by trypsinization, expanded in the continued presence of G418, and assayed for torA expression. All other transfections were performed at least twice and coverslip cultured cells were fixed with formaldehyde at 24 hours post-transfection (plasmid transfections) or between 36 and 48 hours post-transfection (siRNA transfections).

### Plasmid generation and siRNA sequences

The methods and production of torA expression constructs are previously described [10,12]. We generated torA cDNA constructs that lacked residues 22-40 by first using QuikChange PCR mutagenesis to introduce Nhe1 sites between residues 21 and 22, and between 40 and 41, of human torA. We then replaced the sequence between the Nhe1 sites with GFP from pEGFP1 (Clontech). QuikChange was also used to introduce the Y147A mutation. All torA cDNA sequences were fully sequenced following PCR based mutagenesis.

siRNA duplexes were custom synthesized by Invitrogen. Mouse *Tor1aip1 *(LAP1) siRNA duplex sequence begins at basepair 956 in NM_144791 and is previously described [21]. Mouse *Unc84a *(SUN1) siRNA duplex sequence begins at basepair 261 in NM_024451: (GCU AUU GAU UCG CAC AUU A; UAA UGU GCG AAU CAA UAG C) and is matched with control duplex (GCU UAG UCG CUA CAU AUU A; UAA UAU GUA GCG ACU AAG C). Mouse *Unc84b *(SUN2) begins at basepair 2142 in NM_194342: (GCA GGA AGG GAC ACU UCU U; AAG AAG UGU CCC UUC CUG C) and is matched with control duplex (GCA GGA AAC AGU UCG GCU U; AAG CCG AAC UGU UUC CUG C). Mouse *Syne2 *(Nesprin2) begins at basepair 21173 in NM_001005510 and contains sequence from the transmembrane and KASH domain encoding terminal exon of nesprin2: (AUA UAG GUC AUU UAC GUG C; GCA CGU AAA UGA CCU AUA U) and is used with control duplex (GCA AAU GUA CCA AUC GUA UTT; AUA CGA UUG GUA CAU UUG CTT). Mouse *4831426I19Rik *(Nesprin3) siRNA duplex sequence begins at basepair 2732 of NM_172500, which is within the terminal exon that contains the KASH domain sequence: (GCU GUU GCU CCU GCU CUU UTT; AAA GAG CAG GAG CAA CAG CTT) and this is matched with control duplex (GCU UCG UGU CCC UCU GUU UTT; AAA CAG AGG GAC ACG AAG CTT).

### Fluorescent imaging, quantification and statistical analysis

Immunofluorescent labeling of coverslip cultured cells is previously described [21]. Quantification of the relative amounts of NE and ER localized (GFP)torA fluorescence was performed on transfected cells co-labeled with antibodies against GFP and the ER marker Protein Disulphide Isomerase (PDI). We also either colabeled cells, or processed duplicate sets of transfected cells, with antibodies that detect siRNA-targeted proteins to assess the efficiency of target protein knock-down. All images of target proteins were removed prior to assessment of GFP localization to ensure that quantification of (GFP)torA NE/ER fluorescence ratios was conducted blind to both the siRNA treatment and torA isoform.

Labeled samples were examined using a Nikon Eclipse Ti inverted microscope and imaged with Nikon Digital Sight DS-QiMc camera and NIS-elements BR3.0 software. Images of GFP positive cells were captured so that the focal plane visualized the NE. Exposure times were 100 milliseconds or greater and images with saturated signal were excluded to avoid analysis of highly expressing cells. A one-pixel wide measuring line was positioned to bisect the NE and a portion of PDI-labeled ER. The peak of NE localized pixel intensity was used as measure of NE fluorescence. We reduced the possibility of biasing ER fluorescence measurements by consistently determining GFP signal in a region of PDI-positive perinuclear ER. Thus, ER localized GFP signal was calculated as the average intensity of pixels between 0.5 μm to 1 μm distance from the NE. We recorded a 'Background' fluorescence measurement from an untransfected cell within the field and subtracted this value from the NE and ER fluorescence measurements. The intensity of anti-LAP1, SUN1, SUN2, Nesprin2, LAP2, and emerin NE and ER immunofluorescent signals were similarly collected, with the exception that quantification was performed from cells containing either NE- or ER-localized (GFP)torA-ΔE, and background corrections were made from a section of image that lacked adherent cells. A similar procedure was used to determine the ratio of NE/ER localized (GFP)torA-WT fluorescence from verified SUN1 positive or negative cells. In all cases, we used GraphPad prism to analyze the ratios of NE/ER localized fluorescence collected from individual cells, including calculation of mean, s.e.m., Two-Way ANOVA, T-Test, and post-hoc analyses. All graphs show the mean and standard error (s.e.m.) of these values.

### Immunoprecipitations and Western blotting

Confluent dishes of control or (GFP)torA expressing NIH-3T3 cells were collected, pelleted and solubilized in buffer containing 50 mM imidazole-HCl (pH 7.5), 5% glycerol, 0.5% digitonin, 4 mM MgCl_2_, 50 mM NaCl, 1X protease inhibitor cocktail, 2 mM PMSF and nucleotides as indicated. We removed insoluble material by centrifugation at 20, 000 × g and then incubated lysates for 2.5 hour at 4°C with anti-GFP conjugated AminoLink resin (Pierce). We subsequently washed the agarose resin twice with solubilization buffer and eluted captured proteins by 24°C incubation with non-reducing 2X Laemmli buffer. Elutes and lysates were analyzed using standard SDS-PAGE and Western blotting procedures.

### Antibodies

Rabbit anti-GFP and anti-LAP1 antibodies are previously described [21]. We used rabbit polyclonal anti-SUN1 (AbCam), anti-SUN2 (UNC84B) from Sigma and an anti-SUN2 from AbCam. We also used previously described procedures [21] to generate rabbit serum containing polyclonal antibodies against the recombinant luminal domain of SUN1 [30] that we expressed and purified from BL21-RIPL *E. coli *(Stratagene). Anti-Nesprin1 (Abcam), anti-Nesprin2 (Santa Cruz), anti-Nesprin3 (Abcam), anti-emerin (Santa Cruz), anti-LAP2 (Sigma), chicken anti-GFP (AbCam), goat anti-laminA/C (Santa Cruz) and anti-PDI (Assay Designs), are other commercially available antibodies used in this study. Immunofluorescent labeling of cells utilized anti-mouse, chicken or rabbit secondary antibodies conjugated to DyLight 488, FITC or Rhodamine RedX (Jackson Immunoreagents), or AlexaFluor350 conjugated donkey anti-goat (Invitrogen). Primary antibodies used in Western blotting were detected using peroxidase conjugated goat anti-rabbit secondary antibody (Pierce) or an anti-rabbit light-chain specific secondary antibody (Jackson Immunolabs).

## Abbreviations

(AAA+): ATPase associated with various cellular activities; (ER): Endoplasmic Reticulum; (LAP1): Lamina Associated Polypeptide 1; (LULL1): Luminal domain like LAP 1; (LINC): Linker of Cytoskeleton and Nucleus; (NE): Nuclear Envelope; (PDI): Protein Disulphide Isomerase; (torA): TorsinA.

## Authors' contributions

MTJ, DK performed experiments, DYJ performed image analysis, REG designed experiments, analyzed data, prepared data images and wrote the manuscript. All authors read and approved the final manuscript.

## Supplementary Material

Additional file 1 **Figure S1**. LINC complex components are depleted by siRNA transfection of NIH-3T3 cells. Click here for file
