# Supplementary material for: The nuclear envelope localization of DYT1 dystonia torsinA-ΔE requires the SUN1 LINC complex component
Source: BMC Cell Biol. 2011 May 31;12:24. doi: 10.1186/1471-2121-12-24 (PMC3164226; doi:10.1186/1471-2121-12-24)
Supplement: Additional file 1 — Figure S1. LINC complex components are depleted by siRNA transfection of NIH-3T3 cells. [file 1471-2121-12-24-S1.PDF]

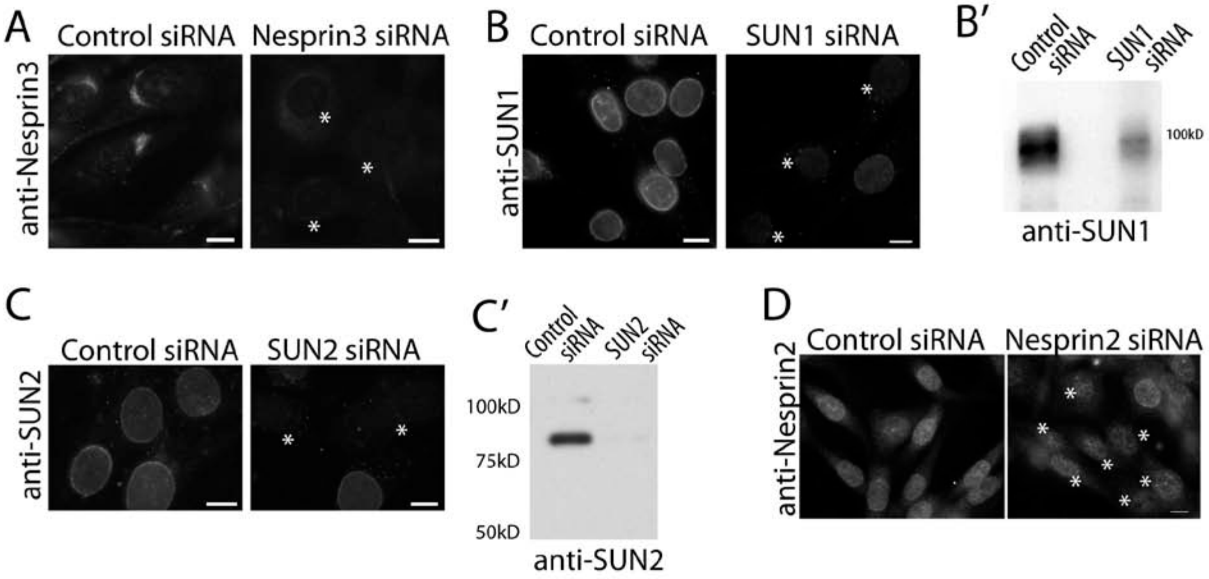

Figure S1

LINC complex components are depleted by siRNA transfection of NIH-3T3 cells.

(A) Nesprin3 is not NE localized in NIH-3T3 cells. Images show anti-Nesprin3 labeling of control or Nesprin3 siRNA transfected cells. All scale bars show 10μm and * symbols highlight siRNA transfected cells that lack LINC complex component immunolabeling.

(B) SUN1 siRNA depletes cellular SUN1 assessed by anti-SUN1 immunofluorescence (B) and Western blotting (B’).

(C) SUN2 siRNA depletes cellular SUN2 assessed by anti-SUN2 immunofluorescence (C) and Western blotting (C’).

(D) Anti-Nesprin2 labeling shows Nesprin2 siRNA depletion of cellular Nesprin2.
